# Efficacy of a multidisciplinary 5‐day headache day‐clinic program: A prospective quasi‐experimental pre–post study in primary headache disorders

**DOI:** 10.1111/head.70133

**Published:** 2026-05-12

**Authors:** Nadja Fiebig, Luise Bartsch, Christine Klötzer, Josephine Gewand, Johanna Ruhnau, Sebastian Strauß, Uwe Reuter, Robert Fleischmann

**Affiliations:** ^1^ Department of Neurology University Medicine Greifswald Greifswald Germany; ^2^ University Hospital Bonn Bonn Germany; ^3^ Department of Neurology Charité‐University Medicine Berlin Berlin Germany

**Keywords:** migraine, multimodal therapy, nonpharmacological intervention, primary headache disorders, quality of life

## Abstract

**Objective:**

To evaluate the effects of a short, structured multimodal day‐clinic program on health‐related quality of life and disability in patients with primary headache disorders.

**Background:**

Although monoclonal antibodies targeting the calcitonin gene‐related peptide (CGRP) pathway have substantially advanced migraine prophylaxis, a large proportion of patients remain insufficiently controlled. Residual disability, comorbidities, and contraindications highlight the need for complementary nonpharmacological approaches. Multimodal programs combining medical, psychological, and behavioral strategies have proven effective in chronic pain but remain insufficiently studied in primary headache populations, particularly in the context of modern pharmacological options.

**Methods:**

In this quasi‐experimental pre–post study at the neurological day clinic of University of Greifswald, we investigated a 5‐day multimodal treatment program delivered by an interdisciplinary team. Patients were recruited between January and July 2021. The intervention comprised medical consultations, psychological therapy, physiotherapy, occupational therapy, structured education, and relaxation training. Patient‐reported outcomes were assessed with the Veterans RAND 12‐Item Health Survey (VR‐12; primary end points: mental component summary [MCS] and physical component summary [PCS]), the Depression Anxiety Stress Scales (DASS‐21), the Headache Impact Test (HIT‐6), and headache diaries. Assessments were performed during the pre‐admission waiting period (V0), at admission (V1), and at 3, 6, and 9 months after treatment (V2–V4). Primary analyses focused on changes from baseline to 3 months, with exploratory analyses for extended follow‐up.

**Results:**

A total of 92 patients were included, most with migraine (83%). The VR‐12 MCS improved significantly over time (*F*[2,162] = 3.23, *p* = 0.042; nominal), increasing from an estimated marginal mean of 36.9 (95% CI, 33.35–40.45) at baseline (V0) to 38.3 (95% CI, 35.54–41.06) at V1, and 41.5 (95% CI, 37.55–45.45) at 3 months (V2). Focusing on the pre‐specified baseline‐to‐3–month contrast, MCS improved from V0 to V2 (mean difference, 4.63; 95% CI, 1.02–8.24; *p* = 0.012; Holm–Bonferroni *p*_adj_ = 0.024). PCS showed no significant changes (*F*[2,162] = 0.95, *p* = 0.387), with estimated marginal means of 38.3 (95% CI, 36.13–40.47) at V0, 37.7 (95% CI, 35.73–39.67) at V1, and 39.0 (95% CI, 36.24–41.76) at V2. Headache frequency did not change significantly across V0–V2 (*F*[2,148] = 0.49, *p* = 0.616). HIT‐6 scores decreased significantly (64.7 ± 3.6 at V0, 64.8 ± 5.0 at V1, and 60.8 ± 5.4 at V2; *F*[2,148] = 5.63, *p* = 0.004). DASS‐21 subscales showed nonsignificant reductions at 3 months, with exploratory analyses indicating gradual improvements at 6 and 9 months.

**Conclusion:**

A short, structured multimodal program in a day‐clinic setting significantly improved the mental component of health‐related quality of life and reduced headache‐related disability in patients with primary headache disorders. Although physical functioning and headache frequency did not change significantly at 3 months, the program provided clinically relevant benefits in coping and mental health. These findings highlight multimodal day‐clinic treatment as a valuable component of comprehensive headache care, even in the era of CGRP‐targeted treatment options.

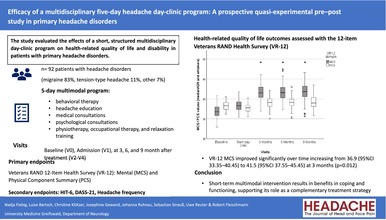

## Plain Language Summary

Although medications have helped many patients with migraine and other headache disorders, there is still a need for other types of treatments. In this study, adults with migraine and other primary headache disorders attended a 5‐day program that combined education, behavioral strategies, physical therapy, and medication review; they completed questionnaires before admission and 3 months after the program. Mental well‐being and headache‐related disability improved, despite minimal changes in physical health scores and headache frequency.

**Abbreviations**
CGRP: calcitonin gene‐related peptide
CI: confidence interval
DASS‐21: Depression Anxiety Stress Scales‐21
HIT‐6: Headache Impact Test‐6
IQR: interquartile range
LMM: linear mixed‐effects model
MCS: mental component summary
PCS: physical component summary
TOST: two one‐sided tests
VR‐12: Veterans RAND 12‐Item Health Survey

## BACKGROUND

Migraine is the leading contributor to the global burden of headache disorders and one of the most disabling neurological conditions worldwide. According to the Global Burden of Disease studies, headache disorders are the second leading cause of years lived with disability, with migraine alone ranking among the top three causes in individuals aged 15–49 years, the most productive years of life.^1^, ^2^ This underlines its major personal, social, and economic impact.

The therapeutic landscape has shifted with the introduction of drugs targeting the calcitonin gene‐related peptide pathway. Monoclonal antibodies (calcitonin gene‐related peptide [CGRP] mAbs) and small molecule antagonists provide targeted prophylaxis with favorable tolerability.^3^, ^4^ Despite these advances, many patients remain insufficiently controlled. Only approximately 30%–40% achieve a ≥50% reduction in monthly migraine days, 10%–15% reach a ≥75% reduction, and optimal control, defined as fewer than 4 monthly migraine days, is achieved in less than half of responders.^5^ Thus, even substantial pharmacological benefit often leaves patients with residual disability and impaired quality of life.^6^ Moreover, treatment can be restricted in specific groups because it is contraindicated (e.g., pregnancy, relevant comorbidities or drug interactions), poorly tolerated despite adequate therapeutic trials, or is not acceptable to the patient after shared decision‐making. Finally, the potential for lasting disease‐modifying effects remains debated.^7^

Migraine is shaped not only by attack frequency but also by unique pathophysiological vulnerabilities. Patients show sensitivity to metabolic and homeostatic stressors such as irregular sleep, circadian disruption, and stress.^8^ Fear‐avoidance behavior, low physical activity, and stigma further increase disease burden.^9^ These dimensions can be addressed by nonpharmacological interventions including aerobic exercise, relaxation and mindfulness techniques, cognitive–behavioral therapy, structured stress management, and education.^10^ Behavioral concepts therefore have substantial leverage to reduce attack frequency, improve coping, and enhance long‐term outcomes.^11^

Guidelines emphasize that such nonpharmacological measures are an integral part of prevention and should be offered both to patients unable to use pharmacological therapy and to those who, despite modern drugs, remain significantly impaired.^5^ However, they cannot be adequately conveyed in routine outpatient consultations, which are brief and medication‐focused.

Multimodal treatment programs provide a structured framework for delivering these strategies.^12^ Although effective in chronic pain,^13^ they are less systematically evaluated in primary headache disorders.^14^ Inpatient programs are resource intensive and disruptive, whereas a day clinic approach is better suited for patients with migraine, who are typically young and ambulatory.^15^ Day clinic programs are cost‐effective, allow structured group‐based interventions, and facilitate transfer into everyday life.^12^

The present study addresses the limited evidence for short multimodal interventions in migraine, in particular in the era of novel highly effective and well‐tolerated pharmacological treatment options targeting the CGRP pathway. We investigate whether a structured 5‐day day clinic program combining medical, psychological, educational, and physiotherapeutic components improves health‐related quality of life in patients with frequent or chronic migraine. The primary hypothesis is that a clinically meaningful improvement in the mental health component summary (MCS) of the VR‐12 persists after 3 months, and we explore whether admission clinical factors are associated with changes in health‐related quality of life in patients with frequent or chronic migraine. We finally explore lasting treatment effects that are sustained at longer‐term follow‐up beyond 3 months.

## METHODS

### Ethics approval and consent to participate

This study was approved by the ethics committee of the Medical Faculty at the University of Greifswald. All participants provided written informed consent before enrollment. All procedures adhered to the Declaration of Helsinki and relevant local regulations.

### Study design and objectives

This prospective quasi‐experimental pre–post study with repeated measures was conducted in outpatients treated at the neurological day clinic, University of Medicine Greifswald, Germany. Recruitment was started in January 2021 and completed in July 2021 by enrolling a consecutive convenience sample. The study was concluded after the last patient reached the final follow‐up evaluation as defined in the protocol. The primary aim was the assessment of the effects of a multimodal treatment program for headache disorders on health‐related quality of life, daily functioning, and disease‐specific outcomes. Assessments were scheduled to start at least 1 month before admission, ensuring a defined pretreatment period without intervention when feasible. However, in a subset of patients admitted on short notice (e.g., expedited/urgent admissions), the pre‐admission assessment (V0) could not always be obtained. In addition, we examined whether admission parameters were associated with subsequent changes in these outcomes. Ethical approval for this study was obtained from the ethics committee of the Medical Faculty at the University of Greifswald (approval no. BB 004/21). Reporting of this nonrandomized evaluation follows the Transparent Reporting of Evaluations with Nonrandomized Designs statement.

### Setting and intervention

This study was conducted at the neurological day clinic, University of Greifswald. Before entering the program, patients remained in routine outpatient care without additional structured interventions, facilitating interpretation of observed changes in relation to the day‐clinic program, although causal attribution remains limited by the nonrandomized design. The day‐clinic program followed a structured weekly schedule combining group‐based and individual sessions. Behavioral therapy comprised four 60‐min group sessions (Monday–Friday) led by a clinical psychologist, focusing on cognitive–behavioral pain coping, stress management, and goal setting.^16^ Headache education was delivered in neurologist‐led seminars covering headache pathophysiology, nonpharmacological strategies, and pharmacological prevention/acute treatment, complemented by nursing‐led input on lifestyle, nutrition, and supporting self‐management skills.^17^ Each patient received an individual medical assessment on day 1 (jointly by physicians and nursing staff), with subsequent ward rounds on the following days to optimize acute and preventive medications. Medication review and optimization were part of routine clinical care and were delivered alongside the educational, behavioral, and physical therapy modules, consistent with integrated multidisciplinary headache management. In addition, patients were offered at least one individual psychological consultation during the week, with further sessions as needed. Physiotherapy consisted of two 1‐hour group sessions emphasizing posture, strengthening, stretching, and light endurance training, preceded by an introduction to physical medicine to tailor exercises and ensure safe prescription.^18^ Occupational therapy included two group sessions addressing ergonomics, coordination, and pacing strategies in everyday and work‐related activities.^19^ Relaxation training (e.g., progressive muscle relaxation) was provided on at least 2 days, and patients were instructed in self‐practice for home use. Adjunctive therapies such as biofeedback, acupuncture, or other stimulation techniques, as well as dance and movement therapy, were available in predefined time slots during the week and are in line with current nonpharmacological migraine approaches.^20^ Across components, patients received written materials, individualized exercise plans, and home assignments to facilitate transfer of cognitive‐behavioral, exercise, and relaxation strategies into daily life. A detailed overview of the weekly schedule, including duration, frequency, and format of all sessions, is provided in Table S1.

### Program standardization and fidelity

To ensure standardization and quality assurance, all therapists followed written protocols specifying session content, sequencing, and learning objectives for each module of the program. The content was aligned within and across professions so that different therapists of the same discipline, as well as medical, psychological, physiotherapy, and occupational therapy staff, conveyed consistent messages and pursued shared treatment goals. The physicians leading the headache program coordinated this alignment, regularly observed selected group and education sessions to monitor adherence to the protocol, and chaired weekly interdisciplinary team meetings in which patient progress and fidelity to the agreed procedures were reviewed.

The program was delivered by licensed health professionals in Germany. Physicians were board‐certified neurologists specialized in headache or neurology residents supervised by respective neurologists. Psychological sessions were led by a clinical psychologist with postgraduate formal psychotherapy training and experience in cognitive–behavioral approaches. Physiotherapy and occupational therapy were provided by state‐licensed therapists with accredited professional training and clinical experience in musculoskeletal and neurologic disorders.

### Participants, recruitment, and eligibility criteria

All patients with a diagnosis of a primary headache disorder who were admitted to the day clinic during the recruitment period were invited to participate. Enrollment was consecutive and based on a convenience sample. Written informed consent was obtained for the collection and analysis of routine treatment data as well as for follow‐up evaluations. No additional study‐specific exclusion criteria were applied beyond routine clinical admission criteria for the day‐clinic program. Admission required an age of ≥18 years, sufficient written and spoken German proficiency to complete questionnaires and participate in group sessions, and the ability to attend the program on‐site on 5 consecutive days (i.e., living within daily commuting distance, approximately ≤50 km).

### Data collection

The evaluation followed a pre–post design with repeated measurements including two pre‐intervention assessments (V0–V1) and post‐intervention follow‐ups (V2–V4). A detailed synopsis of the study plan is presented in Table 1. For primary analyses, V0 served as baseline, since this is the situation when patients seek specialized care, and V2 (3 months) as the prespecified primary end point. Data were collected through patient self‐report using standardized and validated questionnaires. The surveys were embedded into routine clinical documentation, ensuring that they were part of the patient chart and available to both psychologists and physicians for clinical use. Assessments were scheduled at five main time points. The first assessment (V0) was scheduled at least 1 month before admission, during which no structured treatment was provided. This served as a pretreatment observation period to document baseline status before admission to the day‐clinic program; baseline stability was evaluated empirically in the statistical analysis. This included the initial questionnaire as well as sociodemographic information such as age, habits, headache medication, family history, and preexisting conditions. The second assessment (V1) was conducted on the first day of the treatment program. Follow‐up assessments were scheduled by mail after discharge at 3 months (V2), 6 months (V3), and 9 months (V4). Patients were asked to complete the questionnaires at home and return them to the study center.

**TABLE 1:** Study design and assessment schedule.

Domain/Instrument	V0 (≥1 month before)	V1 (day 1 of program)	V2 (3 months ± 6 weeks)	V3 (6 months ± 6 weeks)	V4 (9 months ± 6 weeks)
Sociodemographic data (age, habits, medication, family history, comorbidities)	X				
VR‐12 (health‐related quality of life)	X	X	X	X	X
DASS‐21 (depression, anxiety, stress)	X	X	X	X	X
HIT‐6 (headache‐related disability)	X	X	X	X	X
Headache frequency (days/month)	X	X	X	X	X
Headache intensity (numeric rating scale 1–10)	X	X	X	X	X
Medical history and preexisting conditions	X				
Clinical documentation (psychology/medical chart)		X	X	X	X

*Note*: Assessments were performed at baseline (V0), at admission/start of the interdisciplinary day‐clinic program (V1), and at follow‐up visits at 3 months (V2), 6 months (V3), and 9 months (V4). “X” indicates that the measure was collected at the respective visit. The table summarizes timing of the coprimary outcomes (VR‐12 MCS and PCS) and secondary outcomes (headache frequency and intensity, headache impact, and psychological burden). Visits: V0, baseline before admission; V1, admission/start of program; V2, 3‐month follow‐up; V3, 6‐month follow‐up; V4, 9‐month follow‐up.

Abbreviations: DASS‐21, Depression Anxiety Stress Scales–21; HIT‐6, Headache Impact Test–6; MCS, mental component summary; NRS, numeric rating scale (0–10); PCS, physical component summary; VR‐12, Veterans RAND 12‐Item Health Survey.

### Headache characteristics and questionnaires

The standardized questionnaire set of this study was based on the National Institute of Neurological Disorders and Stroke Common Data Elements and the recommendations of the headache registry of the German Migraine and Headache Society. This ensured standardized data collection, improved quality, and facilitated comparability with related studies.

General health‐related quality of life was assessed using the Veterans RAND 12‐Item Health Survey (VR‐12). This instrument covers physical function, general health, mental health, social function, role limitations due to physical and emotional problems, pain, and activity. It provides two summary scores: the physical component summary (PCS) and the MCS. Scores are standardized to a mean of 50 and a standard deviation (SD) of 10 based on a representative US population sample, allowing comparison across populations and studies. The VR‐12 has been widely validated for use in chronic conditions, including pain and neurological disorders. Moreover, population‐based reference values and mapping have also been established for the German population, providing standardized means and enabling direct comparison in national cohorts.

Headache‐related disability was assessed with the Headache Impact Test (HIT‐6), a brief and widely used instrument that captures the impact of headache on daily activities, social functioning, role limitations, vitality, cognitive functioning, and psychological distress. Scores range from 36 to 78, with higher values indicating greater disability. The HIT‐6 has been validated in migraine and other primary headache disorders and is recommended for both clinical and research use.

Psychological distress was measured with the Depression, Anxiety, and Stress Scale (DASS‐21) short form. This self‐report questionnaire comprises 21 items, with three subscales (depression, anxiety, stress) of seven items each. Items are scored on a 4‐point scale (0–3) referring to the past week. The summed subscale scores are multiplied by two to yield final values, which can be classified into severity categories ranging from normal to extremely severe. In addition, patients reported migraine frequency, expressed as the number of headache days per month, and average headache intensity, measured on an 11‐point numeric rating scale (0–10).

### Statistical analysis

All statistical analyses were performed using the Statistical Package for the Social Sciences (SPSS v29.0, IBM, Armonk, NY). The primary end point was defined as the change in the VR‐12‐Item Health Survey (VR‐12) scores from baseline to 3 months. Both the MCS and PCS scores were analyzed. No statistical power calculation was conducted before the study. The sample size was therefore based on the available data from all eligible patients consecutively admitted to the day clinic during the predefined recruitment period. Secondary end points included changes in headache frequency, headache intensity, and psychological measures assessed by the DASS‐21. These were evaluated not only at 3 months as secondary end points but also explored at extended follow‐up time points to estimate the long‐term trend. Exploratory VR‐12 contrasts at 6 and 9 months were computed nominally for the primary end points to indicate statistical significance in the figures, but were interpreted descriptively. No additional inferential statistics were performed for exploratory end points, as the temporal gap between intervention and assessment was too large to allow causal inference.

Descriptive statistics are presented as mean ± SD unless otherwise stated; for clearly skewed continuous variables, results are reported as median (interquartile range [IQR]); categorical variables are summarized as *n* (%). Distributions of continuous variables were examined using histograms, Q–Q plots, and, where appropriate, Shapiro–Wilk tests. For model‐based quantities derived from linear mixed‐effects models (LMMs), visit‐specific values are reported as estimated marginal means (95% confidence intervals [CIs]), and contrasts are reported as mean differences (95% CIs), unless stated otherwise (e.g., the exploratory two one‐sided test [TOST]‐based equivalence analysis for baseline stability V0–V1 uses 90% CIs). Figures are shown as boxplots to visualize the observed distributions across visits (median/IQR and outliers); inferential conclusions are based on the linear mixed‐effects models.

Longitudinal changes in VR‐12 MCS and PCS, HIT‐6, DASS‐21 subscales, headache frequency, and headache intensity were analyzed using LMMs. For each end point, we specified a model where visit (V0, V1, and V2) was entered as a categorical fixed effect, covariates were prespecified (e.g., age, sex, diagnosis type, and the baseline value of the outcome), there was a subject‐specific random intercept, and finally included the residual error. Alternative covariance structures for within‐subject correlation were compared using Akaike's information criterion (AIC), and an unstructured covariance matrix was selected as providing the best fit. Global time effects were evaluated using type III *F*‐tests from the LMMs, and, where appropriate, pairwise contrasts between visits were derived from the same models. For the exploratory admission‐correlate analyses of VR‐12 MCS change (baseline‐to‐3–month contrast, V0‐V2), we compared a small set of literature‐informed, prespecified nested candidate covariate models using the finite‐sample corrected AIC criterion; no automated stepwise selection procedure was used.

Model assumptions were assessed using histograms and Q–Q plots of residuals, as well as plots of residuals versus fitted values to evaluate homoscedasticity. These did not indicate serious violations for the primary analyses. LMMs were chosen because they make efficient use of all available data and account for incomplete follow‐up under a missing‐at‐random assumption. Consequently, the effective sample size differs by end point and visit, and the denominator degrees of freedom reported with the type III *F*‐tests vary accordingly. VR‐12 MCS and PCS were treated as co‐primary end points. The primary confirmatory comparisons were the baseline‐to‐3–month contrasts (V0 → V2) for MCS and PCS. To control the family‐wise error rate across these two co‐primary tests, *p* values for the V0 → V2 contrasts were adjusted using the Holm–Bonferroni procedure (reported as *p*_adj_). All other *p* values (including omnibus time effects and nonprimary contrasts) are reported as nominal (unadjusted) and interpreted descriptively.

To examine whether the pre‐admission waiting period introduced a clinically relevant change in key patient‐reported outcomes, we conducted an exploratory equivalence analysis for VR‐12 MCS and PCS using the TOST procedure.^21^ VR‐12 summary scores are norm‐based T‐scores with a SD of 10 points in the reference population; we therefore defined an equivalence margin of ±3 points (≈0.3 SD) as the smallest group‐level change considered potentially clinically important. This choice was informed by the health‐related quality‐of‐life literature, where minimal important differences typically lie in the range of approximately 0.3–0.5 SD,^22^, ^23^ and by VR‐12‐specific studies reporting MIDs of approximately 3–6 points for PCS and MCS.^24^ For each outcome, we constructed 90% CIs for the V0–V1 difference; intervals contained within ±3 points were interpreted as not indicating potentially clinically meaningful group‐level change, while recognizing that smaller differences below this threshold cannot be excluded. This equivalence framework was applied only to the pre‐admission waiting period (V0–V1) to assess baseline stability. All analyses were two‐tailed, and a *p* value <0.05 was considered statistically significant.

## RESULTS

### Baseline characteristics

A total of 92 patients were included. Questionnaire availability differed across visits. V1 (first treatment day) data were available for all included patients (*n* = 92). The pre‐admission waiting period assessment (V0) was available for *n* = 75; in the remaining patients, admission occurred on short notice such that V0 could not be completed as scheduled. At 3 months (V2), follow‐up questionnaires were available for *n* = 82 (10 of 92 lost to follow‐up). At 6 months (V3), VR‐12 and HIT‐6 questionnaires were available for *n* = 75 and at 9 months (V4) for *n* = 66. Missing follow‐up data were due to nonattendance at scheduled outpatient follow‐up appointments and/or nonreturn of mailed questionnaires. Headache frequency and intensity values reported below refer to admission/treatment initiation (V1), as these measures were available for all participants. The majority had migraine (76 of 92; 83%), comprising episodic migraine (39 of 92; 42%) and chronic migraine (37 of 92; 40%). Tension‐type headache was present in 10 of 92 (11%), other headache disorders in three of 92 (3%), and diagnosis was missing in three of 92 (3%). Admission headache frequency varied by headache type, with 8.8 ± 4.0 days per month in episodic migraine, 23.7 ± 5.9 days per month in chronic migraine, and 22.5 ± 10.1 days per month in tension‐type headache. Across the cohort, the median admission headache frequency was 14 [IQR 9–22] days per month, whereas average headache intensity was 7.43 ± 1.88 on a 0–10 numeric rating scale. The median number of comorbidities was 1 [IQR 0–2], reflecting the frequent presence of additional diagnoses in this clinical population (see Table 2 for details).

**TABLE 2:** Baseline characteristics of participants at admission/start of the day‐clinic program (V1).

	Unit/category	Total number (%) *n* = 92 (100)
Age, years; mean (SD)	Years, mean ± SD	42.37 ± 13.17
Sex, *n* (%)	Male/female	17 (19)/75 (82)
Headache diagnosis, *n* (%)	Migraine, total	76 (83)
	Episodic migraine	39 (42)
	Chronic migraine	37 (402)
	Tension‐type headache	10 (11)
	Other	3 (3)
	Missing	3 (3)
Medication‐overuse headache, *n* (%)	Yes	25 (27)
	No	67 (73)
Pre‐existing diseases, *n* (%)	Depression	39 (42)
	Anxiety disorder	10 (11)
	Neurological disease	8 (9)
	Psychiatric disorder	9 (10)
	Missing	1 (1)
Smoking, *n* (%)	Yes	21 (23)
	No	58 (63)
	Missing	13 (14)
Alcohol use, *n* (%)	Never	21 (23)
	1/month	26 (28)
	2–4/month	25 (27)
	2–3/week	3 (3)
	4+/week	1 (1)
	Missing	16 (17)
Recreational drug use, *n* (%)	Yes	2 (2)
	No	74 (80)
	Missing	16 (17)

*Note*: The table summarizes sociodemographic and clinical characteristics at baseline. Continuous variables are reported as mean (SD). Categorical variables are reported as *n* (%). “Missing” indicates unavailable information for the respective variable.

Abbreviation: SD, standard deviation.

### Primary end point evaluation and baseline correlates

For the primary end point, the VR‐12 MCS showed a nominal omnibus time effect (*F*[2,162] = 3.225, *p* = 0.042). Consistent with the pre‐specified co‐primary testing strategy, the primary confirmatory comparison was the baseline‐to‐3–month contrast (V0–V2), which demonstrated a clinically meaningful improvement (mean difference, 4.63; 95% CI, 1.02–8.24; nominal *p* = 0.012; Holm–Bonferroni *p*_adj_ = 0.024). The V0–V1 contrast was small and not statistically significant (mean difference, 1.38; 90% CI –0.65 to 3.41; nominal *p* = 0.261), whereas V1–V2 showed improvement (mean difference, 3.25; 95% CI, 0.07–6.43; nominal *p* = 0.045). The VR‐12 PCS did not show evidence of change over time (*F*[2,162] = 0.954, nominal *p* = 0.387), and the co‐primary confirmatory V0–V2 contrast was nonsignificant (mean difference, 0.70; 95% CI, –2.27 to 3.67; nominal *p* = 0.582; Holm–Bonferroni *p*_adj_ = 0.582).

At V2, only the simple model including age and sex showed a statistically significant association with change in VR‐12 MCS (omnibus test χ^2^ = 7.07; *df* = 2; *p* = 0.029). The extended model with admission headache frequency did not provide additional explanatory value (χ^2^ = 2.20; *df* = 3; *p* = 0.532) and the model that further added depressivity and anxiety likewise was not superior to the intercept only model (χ^2^ = 7.55; *df* = 5; *p* = 0.183). We retained the most parsimonious model (age and sex) for the primary interpretation of admission correlates to minimize overfitting and maximize estimate stability given the available sample size. In this preferred model, age was the only admission variable independently associated with change in VR‐12 MCS from baseline (V0) to 3‐month follow‐up (V2) (unstandardized regression coefficient B = –0.028; Wald χ^2^(1) = 7.103; *p* = 0.008), whereas sex was not associated (*p* = 0.626). Fixed‐effect estimates from the intercept‐only, univariable, and final multivariable LMMs are provided in Table S2.

**FIGURE 1:**
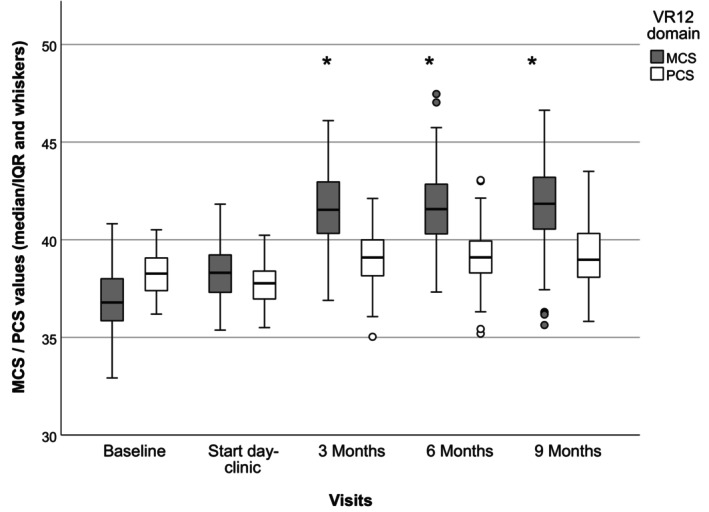
Changes in health‐related quality of life (VR‐12) across study visits. Boxplots of the observed VR‐12 MCS (left) and PCS (right) across visits. The central line indicates the median, the box the IQR, whiskers extend to 1.5 × IQR, and points indicate outliers. Boxplots are shown to display the raw distributions; statistical inference (estimated marginal means/contrasts and *p* values) is derived from the linear mixed‐effects models reported in the Results. Values at 6 and 9 months are displayed to illustrate longer‐term trends. Asterisks indicate nominal baseline‐to‐follow‐up contrasts for VR‐12 MCS, which were V0–V2 *p* = 0.012 (Holm–Bonferroni *p*_adj_ = 0.024); exploratory extended follow‐up contrasts V0–V3 *p* = 0.004 and V0–V4 *p* = 0.003. IQR, interquartile range; MCS, mental component summary; PCS, physical component summary; VR‐12, Veterans RAND 12‐Item Health Survey.

Results are graphically summarized in Figure 1 and furthermore include exploratory time points at 6 and 9 months.

### Secondary end points

Headache frequency did not significantly change between baseline (V0: median, 15 [IQR, 8–28] days/month), treatment initiation/admission (V1: median, 14 [IQR 9–22] days/month) and 3‐month follow‐up (V2: median, 11 [IQR 7–19] days/month; *F*[2,148] = 0.49, *p* = 0.616), indicating no robust changes in headache frequency. HIT‐6 scores were high at baseline (V0: 64.7 ± 3.6) and did not change at treatment initiation/admission (V1: 64.8 ± 5.0). At 3 months (V2), HIT‐6 scores decreased to 60.8 ± 5.4. The overall effect of visit was significant (*F*(2,148) = 5.63, *p* = 0.004). *Post hoc* comparisons confirmed a significant reduction from V0 to V2 (*p* < 0.001), whereas no differences were observed between V0 and V1 or between V1 and V2. For psychological measures (estimated marginal means, 95% CI), depression scores were 11.9 (8.96–14.84) at baseline (V0), 10.8 (7.86–13.74) at treatment initiation/admission (V1), and 9.9 (7.16–12.64) at 3 months (V2). Anxiety scores were 9.9 (7.16–12.64) at V0, 8.3 (5.75–10.85) at V1, and 7.2 (4.85–9.55) at V2. Stress scores were 17.5 (14.36–20.64) at V0, 16.1 (12.96–19.24) at V1, and 14.5 (11.56–17.44) at V2. None of the global models reached significance (depression: *F*[2,148] = 0.46, *p* = 0.631; anxiety: *F*[2,148] = 2.14, *p* = 0.122; stress: *F*[2,148] = 1.54, *p* = 0.217), so no pairwise post hoc tests were performed.

**FIGURE 2:**
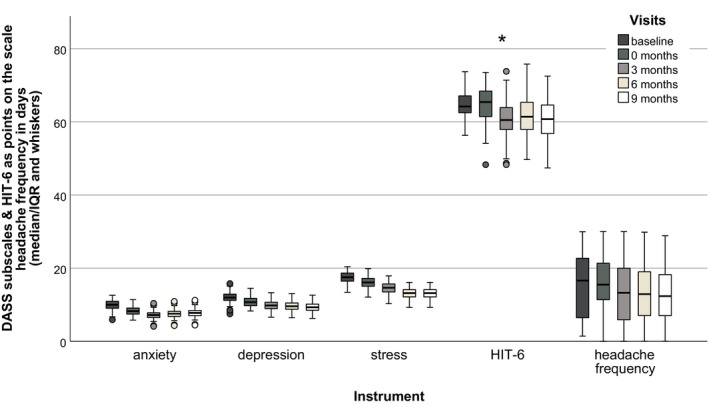
Secondary outcomes: headache frequency, headache impact, and psychological burden. Boxplots of the observed headache frequency (days/month), headache impact (HIT‐6 total score), and psychological burden (DASS‐21 subscales) across visits. The center line denotes the median, the box the IQR, whiskers extend to 1.5 × IQR, and points indicate outliers. Asterisks indicate nominal baseline‐to‐follow‐up contrasts. The plots visualize the raw distributions; statistical inference (global visit effects and, where applicable, follow‐up contrasts/*p* values) is based on linear mixed‐effects models as reported in the Results. For HIT‐6, the overall visit effect was significant (*F*[2,148] = 5.63, *p* = 0.004), and the baseline‐to‐3‐month contrast was highly significant (V0–V2: *p* < 0.001). Extended follow‐up at 6 and 9 months is shown descriptively and causality was not tested for secondary end points for these later time points due to the temporal distance from the intervention. DASS‐21, Depression Anxiety Stress Scales–21; HIT‐6, Headache Impact Test–6; IQR, interquartile range. [Color figure can be viewed at wileyonlinelibrary.com]

Exploratory analyses at extended follow‐up demonstrated that headache frequency continued to decline at both 6 months (V3) and 9 months (V4) compared with baseline, accompanied by further reductions in HIT‐6 scores. The DASS scores for depression, anxiety, and stress, which had shown no significant change at 3 months, exhibited gradual improvements over the longer follow‐up. Results of secondary and exploratory end points are summarized in Figure 2.

## DISCUSSION

In this prospective quasi‐experimental pre–post evaluation, the primary end point, mental health–related quality of life measured with the VR‐12 MCS, improved significantly after the 5‐day multimodal intervention. Physical health (VR‐12 PCS) remained unchanged. Headache frequency did not change robustly at 3 months, yet headache impact (HIT‐6) showed a significant reduction. These findings suggest that participation in the program was associated with improvements in functioning, coping, and mental well‐being rather than attack frequency.

The pattern is consistent with prior multimodal programs that have repeatedly shown stronger effects on quality of life and disability than on headache frequency.^14^, ^25^, ^26^, ^27^, ^28^, ^29^ Our results extend this evidence by showing that even a short 5‐day program can produce meaningful improvements comparable to longer inpatient or outpatient interventions.

### Comparison with inpatient and day‐hospital programs

Previous inpatient and day‐hospital studies have shown that multimodal interventions integrating education, relaxation training, physiotherapy, and psychological therapy reduce disability and improve quality of life in patients with chronic headache.^10^, ^25^, ^28^, ^30^, ^31^, ^32^ Although many of these programs extended over several weeks, our data indicate that a short concentrated 5‐day format can produce similar improvements in mental health–related quality of life and headache impact, although effects on attack frequency were smaller.

Gaul et al.^6^ reported in a 5‐day multidisciplinary program a significant reduction in headache frequency from 13.4 to 8.8 days per month after 12 to 18 months, with 43% of participants achieving a 50% reduction. Wallasch and Kropp^25^ demonstrated a mean reduction in headache days of 5.5 after 6 months and 6.9 after 12 months. Similar benefits have been documented in other tertiary settings.^14^, ^30^ In contrast, our cohort showed no significant reduction in headache frequency at 3 months, although exploratory analyses revealed numerical improvements at 6 and 9 months. At the same time, HIT‐6 decreased significantly at 3 months, underscoring the preferential effect on perceived burden. The discrepancy may be explained by the shorter duration of our intervention and by its behavioral focus, emphasizing coping strategies, psychoeducation, and stress management rather than extended medical optimization.

### Comparisons with pharmacological prevention

Pharmacological preventives, particularly CGRP monoclonal antibodies, have reshaped migraine care by achieving reductions of approximately 3–8 monthly migraine days in phase 3 trials and substantial improvements in patient‐reported outcomes.^33^, ^34^ However, real‐world studies show that only a minority of patients achieve optimal control, commonly defined as ≤4 migraine days per month.^5^, ^6^ Moreover, discontinuation often leads to rapid worsening,^35^, ^36^ and restarting treatment typically restores benefit but confirms the need for ongoing therapy.^37^ Thus, even with modern pharmacological options, residual disability remains.

Against this background, behavioral and nonpharmacological interventions gain relevance as complementary strategies. Absolute effect sizes in our study were smaller than those typically achieved with pharmacological prevention (approximately +3 points VR‐12 MCS and −3 points HIT‐6). Yet the short intervention had no drug‐related adverse effects, is feasible to scale, and addresses domains not targeted by medication, such as coping and mental health. Importantly, nonpharmacological programs should not be considered alternatives but complements that help patients reach higher standards of care, as recommended in the recent IHS consensus on achieving optimal migraine control.^6^

### Admission correlates of change

Exploratory modeling identified age as the only significant predictor, with older patients showing slightly smaller improvements in VR‐12 MCS (see Table S2). The effect was small, approximately 0.3 points per decade or approximately 10% of the overall effect, suggesting limited clinical relevance. Neither sex nor headache frequency nor psychological distress on admission predicted outcome. This suggests that the intervention is broadly applicable across typical day‐clinic populations, and no subgroup could be identified with a low likelihood of benefit. This aligns with prior studies in multidisciplinary settings where predictors of response have been inconsistent.^12^

### Limitations

This study has limitations. First, it was a pragmatic evaluation based on a consecutive convenience sample, and no a priori power calculation was performed. Consequently, the study may have been underpowered to detect small‐to‐moderate clinically meaningful effects—particularly for the coprimary VR‐12 PCS end point—and nonsignificant findings should be interpreted cautiously. Second, the quasi‐experimental single‐center design lacked a concurrent control group during follow‐up. Although the pre‐admission observation period (V0–V1) provided a partial comparator and we used a conservative MID‐based equivalence approach (±3 VR‐12 points; TOST) to assess short‐term baseline stability, we cannot fully separate intervention effects from regression to the mean, spontaneous symptom fluctuation, or the natural course of headache disorders over 9 months. Third, participants were treated in a specialized tertiary headache day clinic and many had complex and treatment‐refractory disease with heterogeneous prior management, which may limit generalizability to primary care or general neurology settings. In addition, changes in preventive medications and concurrent psychological treatment during follow‐up were not systematically captured and may have contributed to outcome changes. Finally, follow‐up missingness (nonreturn of mailed questionnaires) required mixed‐model analyses assuming data were missing at random; attrition‐related bias cannot be excluded, and outcomes beyond 9 months remain unknown.

At the same time, the study has notable strengths. The relatively large consecutive cohort and embedding in routine care enhance validity for tertiary headache services, although generalizability to less specialized settings remains limited. The structured day‐clinic program followed written protocols with predefined session content, sequencing, and learning objectives and was delivered by an interdisciplinary team with specific expertise in headache care, which helped to reduce within‐program variability. Predefined end points, validated instruments, and prospective assessments up to 9 months add robustness to the findings.

## CONCLUSION

A short, structured day‐clinic program improved mental health–related quality of life and reduced headache impact in patients with primary headache disorders. Although effects on headache frequency were modest, the intervention provided meaningful benefits in coping and functioning, supporting its role as a complementary component of comprehensive migraine care.

## AUTHOR CONTRIBUTIONS

**Nadja Fiebig:** Writing – original draft; investigation; visualization; writing – review and editing; data curation. **Luise Bartsch:** Conceptualization; methodology; formal analysis; writing – review and editing. **Christine Klötzer:** Validation; resources; investigation; writing – review and editing. **Josephine Gewand:** Investigation; validation; resources; writing – review and editing. **Johanna Ruhnau:** Resources; investigation; writing – review and editing. **Sebastian Strauß:** Investigation; resources; writing – review and editing. **Uwe Reuter:** Writing – review and editing; resources; supervision. **Robert Fleischmann:** Writing – review and editing; writing – original draft; project administration; resources; supervision; formal analysis; validation; investigation; funding acquisition; visualization; methodology; software; conceptualization.

## FUNDING INFORMATION

This study was not supported by third‐party funding.

## CONFLICT OF INTEREST STATEMENT

**Robert Fleischmann** reports receiving research funding from the German Ministry of Health, German Research Foundation, European Union, Novartis, and TEVA Pharmaceuticals; and received honoraria/consulting fees from AbbVie, Hormosan, Lilly, Lundbeck, Novartis, Organon, and TEVA unrelated to this study. **Uwe Reuter** reports institutional fees from Allergan, AbbVie, Eli Lilly, Lundbeck, Novartis, Pfizer, Medscape, StreaMedUp, Springer, and Teva and research funding from Novartis. **Nadja Fiebig, Luise Bartsch, Christine Klötzer, Josephine Gewand, Johanna Ruhnau,** and **Sebastian Strauß** declare no competing interests.

## Supporting information


Table S1.

## Data Availability

The data sets generated and/or analyzed during the current study are not publicly available due to legal restrictions on data sharing under the EU General Data Protection Regulation (Regulation [EU] 2016/679), but are available from the corresponding author on reasonable request.
