# Supplementary material for: Efficacy of a multidisciplinary 5‐day headache day‐clinic program: A prospective quasi‐experimental pre–post study in primary headache disorders
Source: Headache. 2026 May 12;66(7):1617–27. doi: 10.1111/head.70133 (PMC13327035; doi:10.1111/head.70133)
Supplement: Supplementary file 1 — Table S1. [file HEAD-66-1617-s001.docx]

|  | **Monday** | | **Tuesday** | | | **Wednesday** | | | | **Thursday** | | | **Friday** | | |
| --- | --- | --- | --- | --- | --- | --- | --- | --- | --- | --- | --- | --- | --- | --- | --- |
|  | Arrival | | Arrival | | | Arrival | | | | Arrival | | | Arrival | | |
| **09:00** | Welcome and introduction | | Behavioural therapy group | | | Behavioural therapy group | | | | Behavioural therapy group | | | Behavioural therapy group | | |
| **10:00** | Medical and headache nursing admission interviews  Initial assessment in physical medicine | General counselling with the social worker  **(1)** | Medical ward round | Individual psychological consultations | Acupuncture and other stimulation treatments | Medical ward round | Individual psychological consultations | Acupuncture and other stimulation treatments | Dance and movement therapy | Medical ward round | Individual psychological consultations | Acupuncture and other stimulation treatments | Biofeedback and other stimulation treatments  Individual exercises | | Individual psychological consultations |
| **11:00** |  |  | Medical headache education “Basics” | | |  |  |  |  | Medical headache education “Non-pharmacological therapies” | | | Medical ward round | Acupuncture and other stimulation treatments |  |
| **12:00** | Lunch break | | Lunch break | | | Lunch break | | | | Lunch break | | | Lunch break | | |
| **13:00** | Occupational therapy  Introduction to the therapy program | | Physiotherapy  Strengthening / stretching / light endurance exercise | | | Occupational therapy  Promotion of coordination / ergonomics | | | | Physiotherapy  Strengthening / stretching / light endurance exercise | | | Discharge interviews  Closing session and handing over of documents | | |
| **14:00** | PMR and other relaxation methods | | Biofeedback and other stimulation treatments | | | Medical headache education “Pharmacological therapy” | | | | PMR and other relaxation methods | | |  |  |  |
| **15:15** | Departure | | Departure | | | Departure | | | | Departure | | | Departure | | |

**(1)** individual one-to-one appointments possible during the further course of the week “Headache and diet/nutrition” to be added.

**Supplemental Table 1, Neurology Day Clinic – Weekly Schedule Headache Week.** Standardised weekly schedule of the neurology day clinic programme, showing all treatment modules from Monday to Friday by time of day. The programme includes behavioural therapy groups, medical and nursing admission interviews and ward rounds, individual psychological consultations, headache education sessions, occupational therapy, physiotherapy (strengthening, stretching, and light endurance exercises), social counselling, acupuncture and other stimulation treatments, biofeedback, dance and movement therapy, and relaxation training including progressive muscle relaxation (PMR).

| **Model** | **Predictor** | **B** | **95% CI** | **p-value** |
| --- | --- | --- | --- | --- |
| Intercept-only | Mean MCSchange (V0 to V2) | 4.63 | 1.02 to 8.24 | 0.012 |
| Univariable | Age (per year) | -0.028 | -0.050 to -0.006 | 0.010 |
| Univariable | Sex (binary) | 0.210 | -0.511 to 0.931 | 0.564 |
| Multivariable | Age (per year) | -0.028 | -0.050 to -0.006 | 0.011 |
| Multivariable | Sex (binary) | 0.167 | -0.530 to 0.864 | 0.634 |

**Supplemental Table 2, Associations with VR-12 Mental Component Summary (MCS) change from baseline (V0) to follow-up (V2) estimated using linear mixed-effects models (LMM).** Models include a random intercept for participant to account for within-subject correlation across repeated VR-12 assessments. The intercept-only model provides the estimated mean MCS change in the sample. Univariable LMMs include each candidate predictor separately, and the final multivariable LMM includes age and sex simultaneously (final adjustment set), with all fixed effects shown regardless of statistical significance. Results are reported as fixed-effect estimates (B) with 95% confidence intervals (CI) and two-sided exact p-values. For age, B reflects the expected difference in MCS change per 1-year increase; for sex, B reflects the expected difference in MCS change between sex categories (as coded in the dataset). Analyses include participants with complete data for the outcome and included predictors.

Abbreviations: VR-12, 12-Item Veterans RAND Health Survey; MCS, Mental Component Summary; LMM, linear mixed-effects model; B, fixed-effect estimate; CI, confidence interval.

# TREND checklist for MMTE study

| **TREND item** | **How the requirement is met** | **Manuscript section** |
| --- | --- | --- |
| 1. Title and abstract | The title identifies the intervention, target population and study design; the abstract describes the study design, setting, participants, intervention, outcomes, analysis method and timeframe. A plain language summary follows the abstract. | Title; Abstract |
| 2. Background and objectives | The introduction provides scientific background and rationale for evaluating a 5‑day multimodal day‑clinic programme, citing limitations of pharmacotherapy and the need for non‑pharmacological interventions. Objectives are clearly stated—to assess changes in VR‑12 MCS/PCS, HIT‑6 and DASS‑21 after the programme. | Introduction |
| 3. Participants | Eligibility criteria (routine admission criteria), recruitment methods, setting (neurological day clinic), and recruitment period (Jan–Jul 2021) are described. We also report the number of participants contributing data at the key assessment points (V0 n=75, V1 n=92, V2 n=82). | Methods: Participants, recruitment, and eligibility criteria; Study design and data collection; Results: Baseline characteristics |
| 4. Interventions | The intervention is described in detail: duration (5 days), components (behavioural therapy, education, medical and psychological consultations, physiotherapy, occupational therapy, relaxation), format (group/individual), dosage, protocols and standardisation procedures. | Methods: Setting and intervention; Program standardisation and fidelity; Supplemental Table 1 |
| 5. Objectives and hypotheses | The primary objective is to evaluate changes in mental health‑related quality of life (VR‑12 MCS) after the intervention; secondary objectives involve VR‑12 PCS, HIT‑6 and DASS‑21. Hypotheses are stated implicitly as expected improvements at follow‑up. | Introduction; Methods: Study design and objectives |
| 6. Outcomes | Primary and secondary outcomes are defined, including measurement instruments, timing and units. The primary outcome is VR‑12 MCS at 3 months. Secondary outcomes include VR‑12 PCS, HIT‑6, DASS‑21 subscales and headache frequency/intensity. | Methods: Headache characteristics and questionnaires; Study design and data collection |
| 7. Sample size | No formal power calculation was conducted; all eligible patients during the recruitment period were included (n=92). Rationale for convenience sampling is provided. | Methods: Study design and objectives; Participants, recruitment, and eligibility criteria |
| 8. Randomisation | Not applicable—this is a quasi‑experimental pre–post observational study with repeated measures. | — |
| 9. Blinding | Not applicable—blinding was not feasible in a behavioural intervention. | — |
| 10. Statistical methods | LMMs were used for all inferential analyses, specifying fixed and random effects and covariance structures selected by AIC. Procedures to assess distribution, residual diagnostics and multiple‑testing adjustments are described. | Methods: Statistical analysis |
| 11. Participant flow | Participant flow is reported by stating denominators at each main assessment point and attrition at 3 months. All inferential analyses use linear mixed-effects models so that all available observations contribute to the estimates. | Methods: Study design and data collection; Results: Baseline characteristics; Statistical analysis |
| 12. Recruitment | Recruitment dates (Jan–Jul 2021) and setting (day clinic) are reported. | Methods: Study design and objectives |
| 13. Baseline data | Baseline characteristics are summarised in Table 2, with appropriate measures (mean ± SD, median [IQR], n (%)). Missing data are reported. | Results: Baseline characteristics; Table 2 |
| 14. Baseline equivalence | Comparisons between V0 and V1 indicate no statistically significant differences. An exploratory equivalence test is reported. | Results: Baseline stability (V0 vs V1) |
| 15. Numbers analysed | For each outcome, analyses are based on linear mixed-effects models that include all available observations for that outcome (i.e., participants with incomplete follow-up are retained). Because questionnaire completeness differs across visits and outcomes, effective sample size varies, which is reflected in the model denominator degrees of freedom reported in the Results. | Results: Primary endpoint evaluation; Secondary endpoint evaluation; Statistical analysis |
| 16. Outcomes and estimation | For each outcome, estimated marginal means or medians with 95% CIs (or IQRs) are presented. | Results: Primary and secondary endpoint evaluation; Figures 1–2 |
| 17. Ancillary analyses | Univariable and multivariable models for VR‑12 MCS predictors are presented in Table 4. The time × diagnosis interaction is reported. | Results: Baseline correlates of change; Table 4 |
| 18. Adverse events | As this was a non‑pharmacological behavioural programme, no adverse events were observed or reported. | Discussion: Interpretation/limitations (intervention safety) |
| 19. Interpretation | The discussion interprets results in light of the study design and existing literature, considers alternative explanations, and discusses public health implications. | Discussion |
| 20. Generalisability | The limitations section discusses generalisability, noting the single‑centre convenience sample and the need for multicentre controlled studies. | Discussion: Limitations |
| 21. Overall evidence | The conclusion summarises findings and emphasises that multimodal day‑clinic programmes may complement standard care but require further evaluation in controlled trials. | Conclusion |
